# Supplementary material for: Predicting the potential global distribution of Ixodes pacificus under climate change
Source: PLoS One. 2024 Aug 27;19(8):e0309367. doi: 10.1371/journal.pone.0309367 (PMC11349213; doi:10.1371/journal.pone.0309367)
Supplement: Attachment — Submitted filename: Response to Reviewers.docx [file pone.0309367.s007.docx]

Dear Editor,

Thank you very much for your advice on our manuscript (Manuscript ID: PONE-D-24-17968) entitled “Predicting the Potential Global Distribution of Ixodes pacificus under climate Change”. We also thank the academic editor and reviewers for their constructive comments and suggestions. We have revised the manuscript accordingly, and all amendments are indicated in the file labeled 'Revised Manuscript with Track Changes'. In addition, our point-by-point responses to the comments are listed below this letter.

We hope that our revised manuscript is now acceptable for publication in your journal and look forward to hearing from you soon.

With best wishes,

Yours sincerely,

Fengfeng Li, PhD

Shandong Second Medical University, China

**The response for** **the academic editor and reviewers:**

First of all, we would like to express our sincere gratitude to the academic editor and reviewers for their constructive and positive comments.

1. In your Methods section, please provide additional information regarding the permits you obtained for the work. Please ensure you have included the full name of the authority that approved the field site access and, if no permits were required, a brief statement explaining why

Response: Thank you for your insightful suggestion. I ensure that my method section already includes the full name of the authority that approved the field site access and, I quoted them in the manuscript at their request. Other information parts that are not indicated for work permits are free and open sites.
